# Eco-friendly flower-like ZnO nanostructures for photocatalytic treatment of levofloxacin-contaminated wastewater

**DOI:** 10.1038/s41598-026-55305-2

**Published:** 2026-05-27

**Authors:** Raja Selvaraj, Stuthi A. Shetty, Sujesh Sudarsan, Thivaharan Varadavenkatesan, Ramesh Vinayagam

**Affiliations:** Manipal Institute of Technology, Manipal Academy of Higher Education, Manipal, India

**Keywords:** Rubber fig leaves, Zinc oxide nanoflowers, Green synthesis, Photocatalytic degradation, Levofloxacin removal, Chemistry, Environmental sciences, Materials science, Nanoscience and technology

## Abstract

This work reports an environmentally benign strategy for producing zinc oxide (ZnO) nanoflowers from rubber fig (*Ficus elastica*) leaf extract and assesses their efficacy in sunlight-assisted degradation of levofloxacin. UV-visible spectrum displayed a clear absorption band at 373 nm, characteristic of ZnO nanoparticles. Field-emission scanning electron microscopic images showed flower-like nanostructures, and Fourier transform infrared spectra showed phytochemical functional moieties from the leaf extract that assist nanoparticle formation and stabilize the surface. X-ray diffraction patterns endorsed the crystallinity with a mean crystallite dimension of 44.86 nm and lattice parameters matching the hexagonal wurtzite structure. X-ray photoelectron spectroscopy further verified Zn^2+^ through Zn 2p3∕2 and Zn 2p1∕2 peaks at 1021.32 and 1044.42 eV, a lattice oxygen signal at 529.9 eV, and minor C 1s contributions from residual phytochemicals, indicating high purity and a structurally stable lattice. Photoluminescence spectra showed a near-band-edge ultraviolet emission at 403 nm together with visible bands associated with defect-related states. Under natural sunlight, the ZnO nanoflowers consistently degraded levofloxacin, and the removal improved as the catalyst dose increased. The dataset follows a pseudo-first-order expression; the estimated rate constants span 0.0044 to 0.0056 min^− 1^ and R^2^> 0.99. The rise in performance at higher loadings aligns with a larger number of accessible surface sites and more efficient formation of reactive oxygen species. Collectively, ZnO nanoflowers synthesized by a green route show strong promise for the sustainable treatment of pharmaceutical wastewater.

## Introduction

Water pollution is a major environmental concern because industrial, municipal, and agricultural discharges introduce a wide range of contaminants into aquatic systems. Among these, persistent dyes, toxic metal species, and pharmaceutical residues are particularly concerning because they remain in water for long periods and endanger both ecosystem integrity and human health. Dyes are often toxic and poorly biodegradable, while many metal species are highly mobile, carcinogenic, and difficult to remove using conventional treatment methods^1,2^. Within the group of pharmaceutical pollutants, antibiotics deserve special attention because of their essential role in human and veterinary medicine^3^. However, their extensive and often poorly regulated use, combined with their incomplete absorption in living organisms and poor biodegradability^4^, has led to the continuous discharge of antibiotic residues into rivers, lakes, soil, and wastewater treatment plant effluents. Once released, these residues can persist in the environment and interact with aquatic organisms, thereby exerting selective pressure on microbial population and promoting the spread of antibiotic-resistant strains and resistance genes^5^. As a result, antibiotics that were once highly effective are becoming less reliable, common infections are increasingly difficult to treat, and the burden on healthcare systems continues to grow.

Levofloxacin (LVX) is a fluoroquinolone that is widely prescribed and active against a broad spectrum of Gram- negative and positive pathogens. It is of particular concern because it is chemically stable, heavily used, and strongly bioactive. In humans, only part of the administered dose is metabolized, while the remainder is excreted largely unchanged. This unmetabolized fraction can accumulate in surface waters, groundwater, and wastewater treatment plant effluents, where it exerts continuous ecological pressure and shifts microbial communities toward reduced antibiotic susceptibility^6^. Hence, there is an increasing interest in treatment methodologies that degrade LVX effectively.

Among the different routes available for treating persistent pharmaceutical residues, advanced oxidation processes are now widely explored. Within this group, photocatalytic degradation has become a potential green method and has also attracted broader interest in clean energy-related applications^7^. Techniques such as electrochemical oxidation, photo Fenton systems, ozonation, and heterogeneous photocatalysis generate highly reactive species that can attack and, in many cases, mineralize complex organic molecules^8^. Sunlight-driven semiconductor photocatalysis is especially appealing because it has high oxidative potential with an abundant renewable energy source. When a suitable photocatalyst is illuminated, electron and hole pairs are created and initiate a sequence of redox reactions that progressively degrade the target contaminant^9^. The overall performance strongly depends on the photocatalyst nature, in particular its optical absorption, crystal structure, and surface chemistry.

Zinc oxide (ZnO) is a popularly explored semiconductor for environmental remediation because it combines low cost, earth abundance, reduced toxicity, and good chemical and thermal stability^10^. It has a band gap of about 3.37 eV with high exciton binding energy of 60 meV, which supports its strong photoactive behavior^11,12^. While exposed to light irradiation, ZnO can produce reactive oxygen species (ROS), which are crucial to degrade persistent organic contaminants^13^. Another important advantage is that ZnO can be synthesized readily through simple aqueous and plant-mediated routes, while its morphology and crystallinity can be tailored by varying the preparation conditions^14^. It also serves as a versatile platform for further enhancement through doping, carbon coupling, and heterojunction design^15^.

However, many conventional ZnO synthesis methods rely on high temperature treatment, organic solvents, and strong reducing or capping agents, which partly diminish the environmental benefits of the final material^16^. Plant-based green synthesis offers a more sustainable option. In such systems, phytochemicals naturally present in leaves, stems, or fruits act simultaneously as reducing and stabilizing reagents, allowing nanoparticle formation in water without additional toxic reagents^17,18^. A range of plant species have been reported in the literature, including *Epipremnum aureum*^19^, *Ocimum basilicum*^20^, *Clerodendrum infortunatum*^21^, and *Justicia adhatoda*^22^. Although the resulting ZnO materials perform well in dye removal and show clear antibacterial activity, their application to persistent pharmaceutical pollutants remains less explored, particularly for fluoroquinolone antibiotics. This gap motivates testing additional plant extracts that can yield stable and efficient ZnO nanostructures suited to sunlight-driven photocatalytic treatment of pharmaceutical residues.

In this context, *Ficus elastica*, commonly called rubber fig or rubber tree, is a *Moraceae* species that is widely distributed in tropical climates. Its leaves are rich in tannins, phenolic compounds, and phytosterols^23^, making the aqueous extract a suitable green reagent for metal oxide nanostructure formation. The novelty of the present work lies in the synthesis of ZnO nanoflowers with *F. elastica* leaf extract and in demonstrating its photocatalytic activity toward LVX degradation under natural sunlight, which, to the best of our knowledge, has not yet been examined.

Accordingly, this study investigates the sunlight-driven degradation of LVX using rubber fig-derived ZnO nanoflowers and systematically evaluates the synthesized material through UV-visible spectroscopy, FESEM and EDS, X-ray diffraction, FTIR, XPS, BET surface area analysis, and photoluminescence spectroscopy. The effect of catalyst dosage on photocatalytic performance was also examined, and the resulting concentration profiles were fitted with pseudo-first-order kinetics. Taken together, the plant-mediated synthesis combined with natural sunlight as the irradiation source supports rubber fig-derived ZnO nanoflowers as a practical photocatalyst for the remediation of pharmaceutical wastewater.

## Raw materials and methodologies used

### Raw materials

The rubber fig leaves were gathered from the Manipal College campus, India, and used to prepare the rubber fig leaf extract (RFLE). Zinc acetate dihydrate (Merck, India) served as the precursor for nanoparticle synthesis, and sodium hydroxide (Merck, India) was used as the precipitating agent. LVX (> 99%) was procured from Yarrow Chem Products, Mumbai, India, and employed as the model pharmaceutical pollutant in the photocatalytic experiments.

### Synthesis of RFL-ZnO nanoflowers

**Fig. 1:**
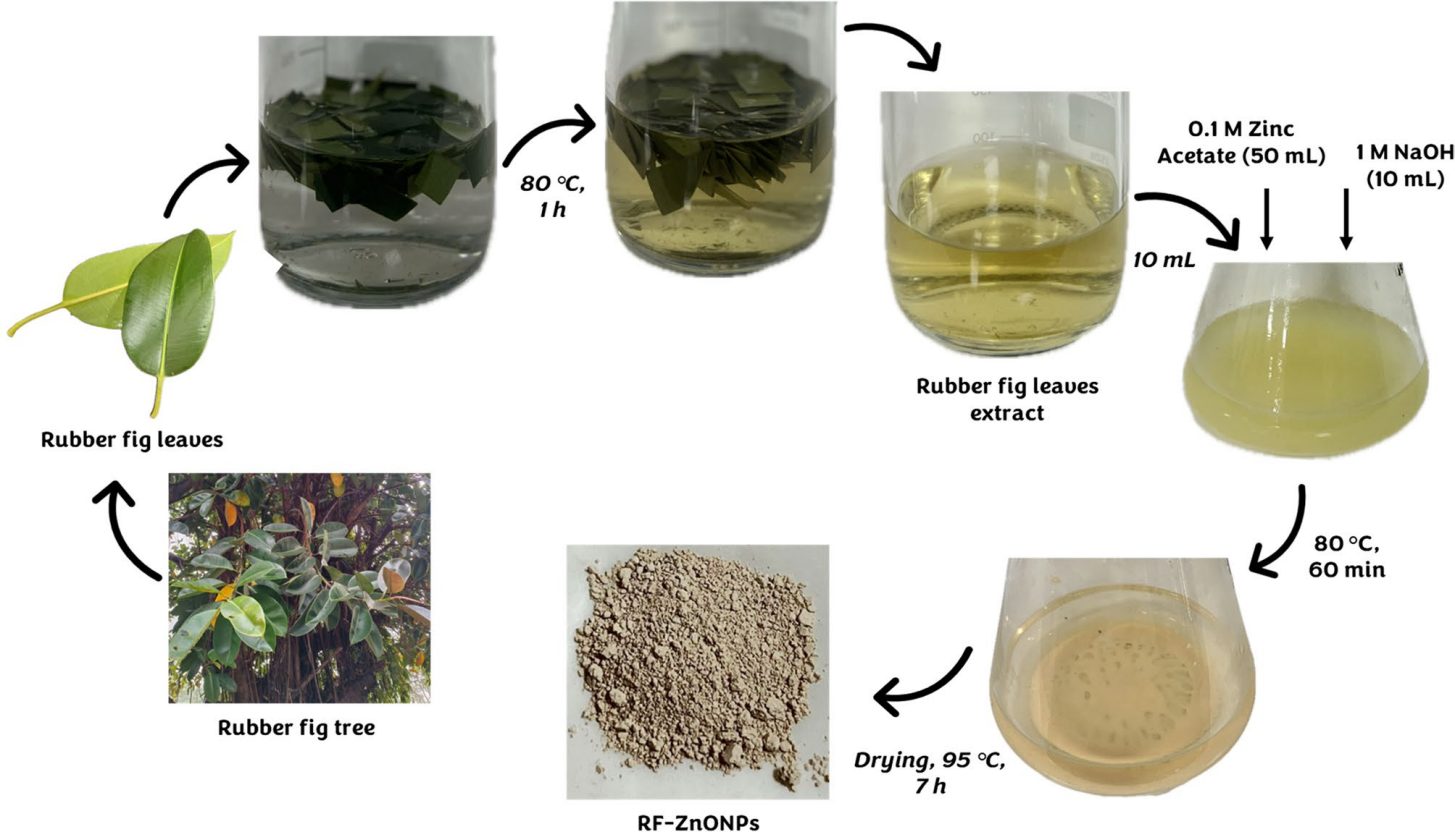
Schematic representation of RFL-ZnO nanoflower synthesis process.

Rubber fig leaf pieces were mixed with distilled water at a 1:10 g/mL ratio for RFLE preparation. The contents were heated (80 °C, 1 h, water bath) to facilitate the release of water-soluble phytochemicals. Post-cooling, the contents were filtered to obtain the extract RFLE. ZnO nanoflowers were prepared by adding 10 mL of RFLE and 10 mL of 1 M NaOH to 50 mL of 0.1 M zinc acetate dihydrate in a flask. The contents were kept in a magnetic stirrer (80 ℃, 1 h) to favour nanoparticle formation. The obtained products were then centrifuged, distilled water-washed till neutral pH, and oven-dried (95 ℃, 7 h). The dried mass was stored in an airtight container and designated as RFL-ZnO (Fig. 1).

### Characterization techniques

UV-visible spectrophotometry was used to examine the optical absorption characteristics of the RFL-ZnO nanoflowers. The optical band gap energy was calculated from the UV-visible absorption data using the *Tauc* relation for a direct allowed transition. The morphology was inspected with field-emission scanning electron microscopy (FESEM, Neon 40, Carl Zeiss). Elemental nature was examined with energy dispersive X-ray spectroscopy (EDS, Oxford Instrument, UK). Crystal structure was analysed with X-ray diffraction (XRD, D8 Advance, Bruker, Germany). The specific surface area was calculated using Brunauer-Emmett-Teller (BET) method (Smart Instruments, Mumbai). The functional moieties from the plant extract were probed with Fourier transform infrared spectroscopy (FTIR, Shimadzu-8400 S). Oxidation states were examined by X-ray photoelectron spectroscopy (XPS, K-α, Thermo Fisher Scientific, UK). Photoluminescence emission was recorded with a fluorescence spectrometer (PL, V-730, JASCO, UK).

### Photocatalytic potential of RFL-ZnO

The photocatalytic potential of the RFL-ZnO nanoflowers was assessed by degrading LVX under natural sunlight. 30 mg/L LVX solution was used, and tests were run at catalyst loadings of 0.05, 0.10, and 0.15 g/L. For each run, 100 mL of the contents was kept in a flask and mixed in the dark (30 min) to allow the antibiotic to equilibrate with the catalyst surface. The flasks were then exposed to direct sunlight with continuous stirring so that the particles remained well suspended. Experiments were done between 11.00 a.m. and 3.30 p.m, during February 2025, a window with relatively strong and stable sunlight. Samples were withdrawn at regular time intervals, filtered immediately to remove the catalyst, and analysed for the residual LVX. Concentrations were measured with a Shimadzu UV 1900i at 287 nm. Each condition was tested in triplicate, and mean values were used for subsequent analysis to ensure reliability and reproducibility.

The degradation potential was calculated using Eq. (1):

1 $$\:\mathrm{Degradation}\:\left({\%}\right)=100\:\times\:\:\frac{{\mathrm{C}}_{0}-{\mathrm{C}}_{\mathrm{t}}}{{\mathrm{C}}_{0}}$$

where $\:{C}_{0\:}$ and $\:{C}_{t}\:$denote the respective initial and time-specific sunlight-irradiated LVX concentration. The photocatalytic degradation kinetic data were analyzed with a pseudo-first-order model, given by Eq. (2):

2 $$\:ln\left(\frac{{C}_{0}}{{C}_{t}}\right)=kt$$

where *k* denotes the rate constant for LVX degradation under sunlight.

Reactive species trapping experiments were performed to find the significant oxidizing species accountable for LVX degradation, following a modified procedure based on a previously reported methodology^24^. Briefly, 15 mg of RFL-ZnO was added to 30 mg/L of 100 mL LVX solution and kept for 30 min, stirring to attain adsorption-desorption equilibrium. Thereafter, Disodium ethylenediaminetetraacetate (EDTA-2Na, 5 mM), Isopropanol (IPA, 10 mM), Silver nitrate (AgNO_3_, 100 mM), and p-benzoquinone (PBQ, 1 mM) were added individually as scavengers for holes (h^+^), hydroxyl radicals (•OH), electrons (e^−^), and superoxide radicals (•O_2_^−^), respectively. The suspensions were then exposed to natural sunlight under continuous stirring. After the reaction period, aliquots were collected, centrifuged, and the residual LVX concentration was measured. A control experiment was done without scavenger addition.

### Reusability studies

The reusability of RFL-ZnO nanoflowers was assessed under the optimized photocatalytic conditions using a modified procedure based on a previously reported protocol^25^. In each cycle, 15 mg of RFL-ZnO was added to 100 mL of 30 mg/L LVX solution and exposed to natural sunlight. At the end of each cycle, the catalyst was recovered by centrifugation, washed sequentially with distilled water, ethanol, and again distilled water, and then dried for 12 h before reuse. The percentage degradation in each cycle was calculated using Eq. (1), and the performance retained in subsequent cycles relative to the first cycle was used to examine stability and regeneration.

## Results and discussions

### UV–visible spectral analysis of RFL-ZnO nanoflowers

**Fig. 2:**
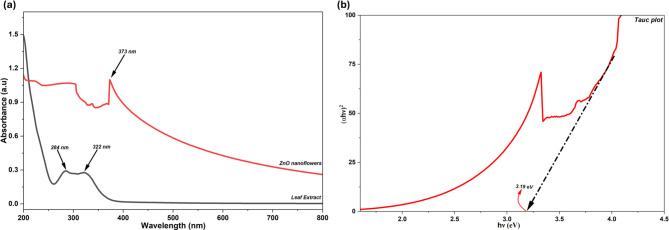
**(a)** UV–Vis absorption spectra of RFLE and RFL-ZnO nanoflowers, and **(b)** Tauc plot of RFL-ZnO nanoflowers for estimation of the optical band gap energy.

The optical response of the RFLE and the resulting RFL-ZnO nanoflowers were examined by UV-visible spectroscopy (Fig. 2a). During the reaction, the colour of the contents changed from yellow to off-white, providing an initial visual indication of particle formation, which was later supported by the appearance of a distinct absorption peak. The leaf extract showed two small peaks at 284 nm and 322 nm. These features are consistent with phytochemicals like flavonoids and phenolic compounds as reported earlier^26,27^. Literature on *F. elastica* also indicates the presence of polyphenols, alkaloids, saponins, flavonoids, and tannins^28^. In the present system, these phytochemical classes are considered to function primarily as complexing, nucleation-directing, capping, and stabilizing agents by interacting with Zn^2+^ species through hydroxyl and carbonyl functionalities, thereby assisting controlled growth and limiting agglomeration of the RFL-ZnO nanoflowers. A comparable role has been reported in a previous nanoparticle synthesis study using *F. elastica* leaf extract, where phytochemical classes such as alkaloids, flavonoids, terpenoids, aldehydes, ketones, and amides were described as reducing, antioxidant, and capping constituents that facilitate nanoparticle formation and help prevent agglomeration^29^.

The RFL-ZnO nanoflowers showed a separate absorption maximum at 373 nm, characteristic of ZnO and attributed to the near-band-edge electronic transition. The shift in absorption from the extract to the ZnO sample provides direct evidence of successful nanostructure formation. Comparable UV-Vis absorption behavior has been reported for green-synthesised ZnO, with maxima at 372 nm for *Lepidagathis ananthapuramensis*^30^, 373 nm for *Merremia quinquefolia*^31^, and 375 nm for *Carica papaya* leaf extract^32^. To further evaluate the optical features, the band gap energy was estimated using the UV-visible absorption dataset with the *Tauc* relation for a direct allowed transition, which gave an extrapolated band gap value of 3.19 eV (Fig. 2b). This value is within the range documented for green synthesized ZnO derived from plant and biomass sources. For comparison, ZnO marigold-shaped nanostructures synthesized using the leaf extract of *Eucalyptus globulus* showed a band gap of 3.05 eV^33^, and ZnO synthesized using *Lepidagathis ananthapuramensis* leaf extract showed values in the range of 2.81 to 3.01 eV^30^.

### Morphological, elemental, textural properties and growth mechanism of RFL-ZnO nanoflowers

**Fig. 3:**
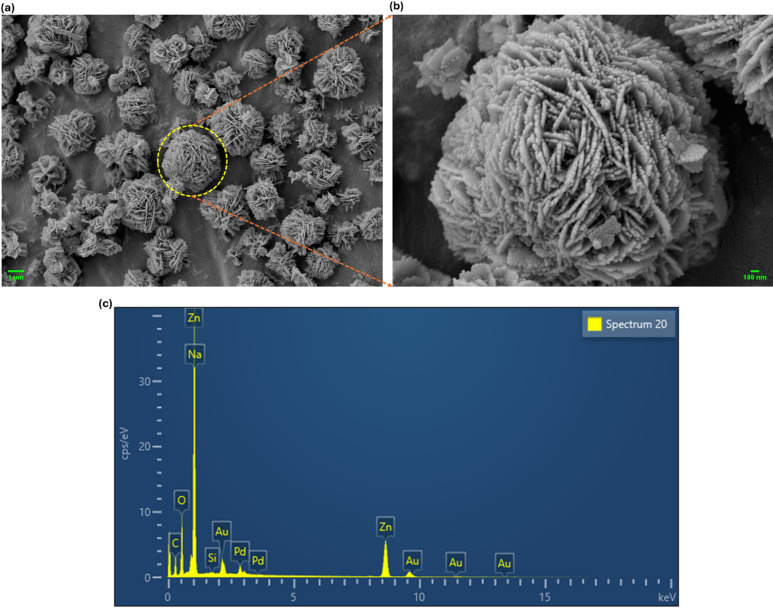
**(a)** Low magnification FESEM image and **(b)** high magnification FESEM image of RFL ZnO nanoflowers; **(c)** EDS spectrum of RFL-ZnO nanoflowers showing dominant Zn and O signals (Si likely originates from the substrate, Na may be due to residual base, and Au and Pd arise from the conductive coating used for FESEM analysis).

The FESEM images (Fig. 3a and b) show that the RFL-ZnO forms clear flower-like assemblies that resemble Marigolds. At lower magnification (Fig. 3a), these flowers appear uniformly distributed and are formed from many closely packed spherical particles. At higher magnification (Fig. 3b), the agglomeration of these assembled structures becomes more evident. Each nanoflower is composed of numerous petal-like features made of densely packed nano rods or nano sheets radiating from a central core^34^. The formation of these nanoflowers occurs through a stepwise process. Initially, individual nanopetals are produced. Due to the Coulombic attraction between the polar surfaces of ZnO, these nanosheets aggregate and self-assemble into flower-like clusters. High incubation temperature and the concentration of Zn^2+^ and OH^−^ ions play a crucial role in directing this growth^35^. At higher reactant concentrations, homogeneous nucleation occurs, leading to supersaturation and aggregation of ZnO nuclei. To reduce surface energy, the sheets gather toward a common center that becomes the flower core, and their mutual angles adjust with temperature through van der Waals forces^36^. Since spherical structures have the lowest surface energy, multiple nanosheets arrange themselves radially to reduce the overall interfacial energy, which results in the marigold-like RFL-ZnO nanoflowers. Such flower-like structures and their gentle agglomeration provide a high accessible surface area, which benefits adsorption and photocatalysis^37^. Similar morphologies have been reported for green routes using *Eucalyptus globulus*^33^, *Leucophyllum frutescens*^38^, and *Justicia adhatoda* leaf extracts^22^. EDS spectra (Fig. 3c) show strong Zn and O signals that confirm ZnO^22^, with minor C, Si, Na, Au, and Pd peaks. Among these, Au and Pd arise from the conductive coating used during FESEM sample preparation, Si likely originates from the substrate, and Na may be attributed to residual base.

Nitrogen adsorption–desorption measurements gave a BET specific surface area of 23.15 m^2^/g and a total volume pores of 0.0589 cm^3^/g. The BJH average pore size was 10.18 nm, consistent with a mesoporous texture. This pore network supports photocatalysis by improving the adsorption of reactants and allowing effective transport during photodegradation.

### XRD studies

**Fig. 4:**
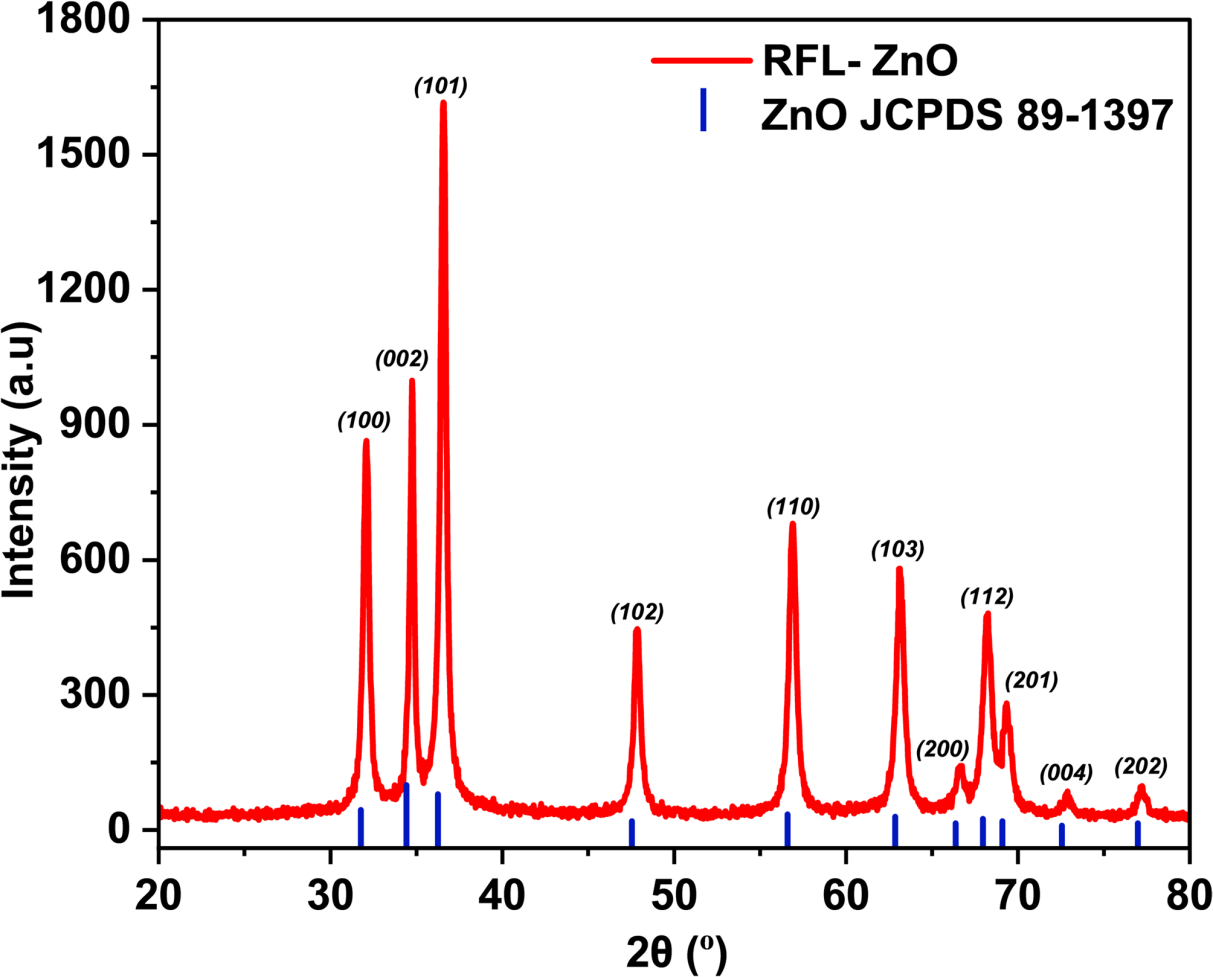
XRD pattern of RFL-ZnO along with the reference JCPDS card of hexagonal wurtzite ZnO.

The distinct and sharp XRD diffraction signals (Fig. 4) appear at 2θ values of 32.08°, 34.75°, 36.57°, 47.81°, 56.89°, 63.13°, 66.67°, 68.21°, 69.34°, 72.86°, and 77.21°. These reflections are indexed to the respective planes of (100), (002), (101), (102), (110), (103), (200), (112), (201), (004), and (202), and align satisfactorily with the standard hexagonal wurtzite ZnO structure reported in JCPDS 89-1397^39^. The most intense 36.57° signal, assigned to the (101) plane, indicates a preferred orientation along this direction. The lack of additional diffraction signals related to impurity phases confirms the purity of the material within the detection limit of the instrument.

The calculated lattice factors are a = 0.3222 nm and c = 0.5163 nm, which are slightly lower than the standard (a = 0.3249 nm and c = 0.5206 nm)^40^. This small contraction is consistent with lattice strain and finite-size effects^41^, and may also reflect weak surface interactions with phytochemicals during green synthesis. However, the unchanged c/a ratio (= 1.602) indicates that the hexagonal structure remains stable.

The average crystallite size, determined from the Scherrer equation using the major diffraction peaks, was 44.86 nm which is comparable with the literature for ZnO nanoparticles synthesized using cetyltrimethylammonium bromide (47.12 nm)^42^ and *Piper betle* leaf extract (36 nm)^43^. The experimental d-spacing of 2.46 Å for the (101) plane is close to the standard value of 2.48 Å^32^, further confirming structural integrity. Together, these findings confirm that the biomolecules of the RFLE play as stabilizing agents and do not alter the crystal structure of the synthesized ZnO.

### XPS analysis

**Fig. 5:**
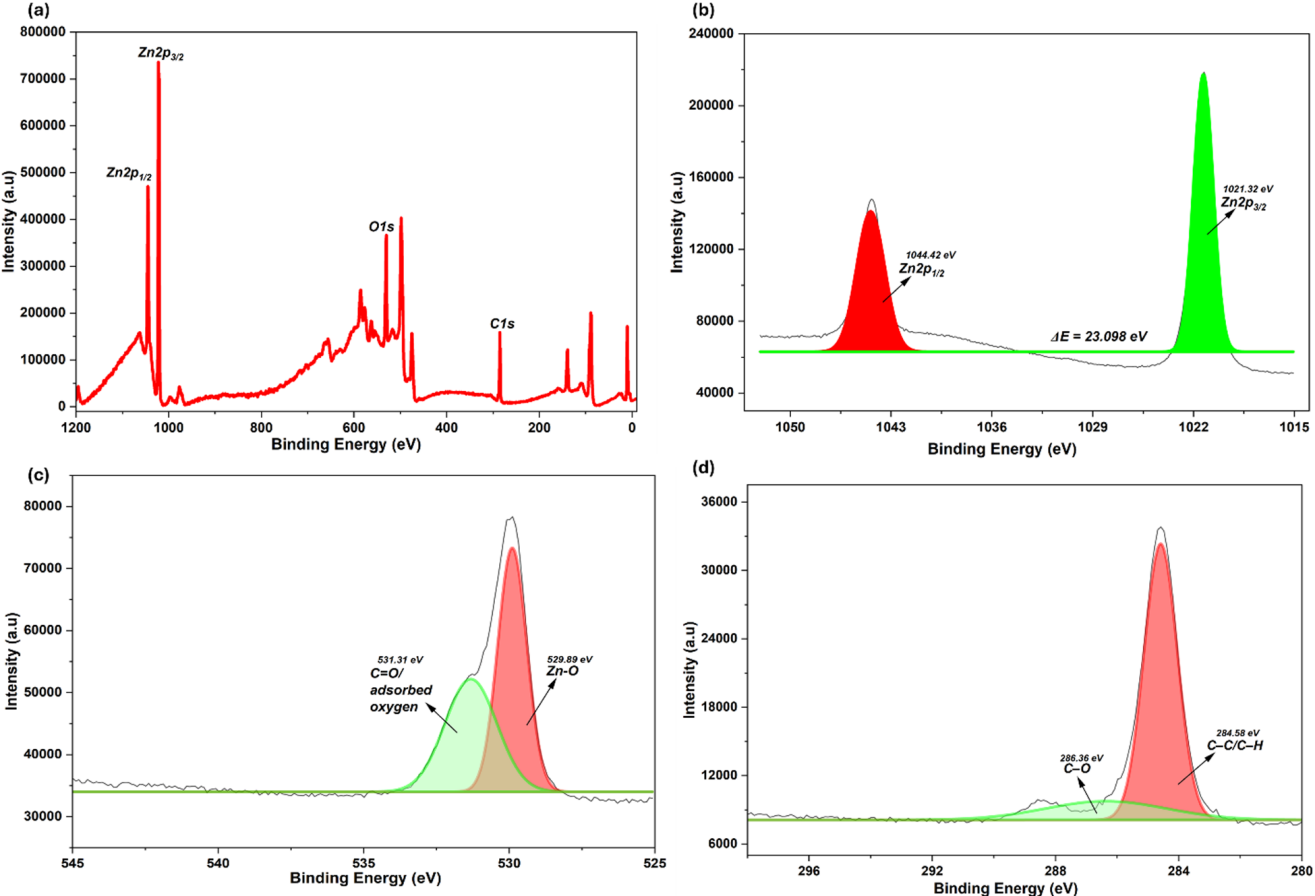
XPS spectra RFL-ZnO Nanoflowers: wide scan (**a**), Zn2p region (**b**), O1s region (**c**), and C1s region (**d**).

Figure 5a shows the wide scan XPS spectrum of RFL-ZnO nanoflowers. Strong signals for Zn 2p, O 1s, and C 1s confirm zinc and oxygen as the important constituents with a small amount of surface carbon, and the absence of additional features indicates no detectable inorganic impurities. The deconvoluted Zn 2p spectra in Fig. 5b displays a clear doublet with signals at 1021.32 eV for Zn 2p3/2 and 1044.42 eV for Zn 2p1/2. The spin-orbit separation of 23.10 eV, together with the observed binding energies, is consistent with Zn^2+^ in the wurtzite ZnO lattice and rules out metallic zinc. These findings are coherent with earlier studies on ZnO synthesized with leaf extracts of *Cinnamomum tamala*^44^ and *Carica papaya*^32^.

The O1s spectrum (Fig. 5c) exhibits a dominant signal at 529.89 eV, corresponding to lattice oxygen (O^2−^) coordinated with Zn^2+^ ions in the ZnO crystal structure^30^. A weaker shoulder at 531.31 eV is also present and is commonly attributed to oxygen vacancy-related species or to surface adsorbed oxygen^45^. The C 1s spectrum in Fig. 5d features a main signal at 284.58 eV that reflects C-C or C-H bonds originating from organic constituents of the leaf extract^46^. A smaller 286.36 eV peak corresponds to C-O functionalities, indicating that certain phytochemicals remain on the ZnO surface and likely assisted nanoparticle stabilization during synthesis^47^.

### PL spectral analysis

**Fig. 6:**
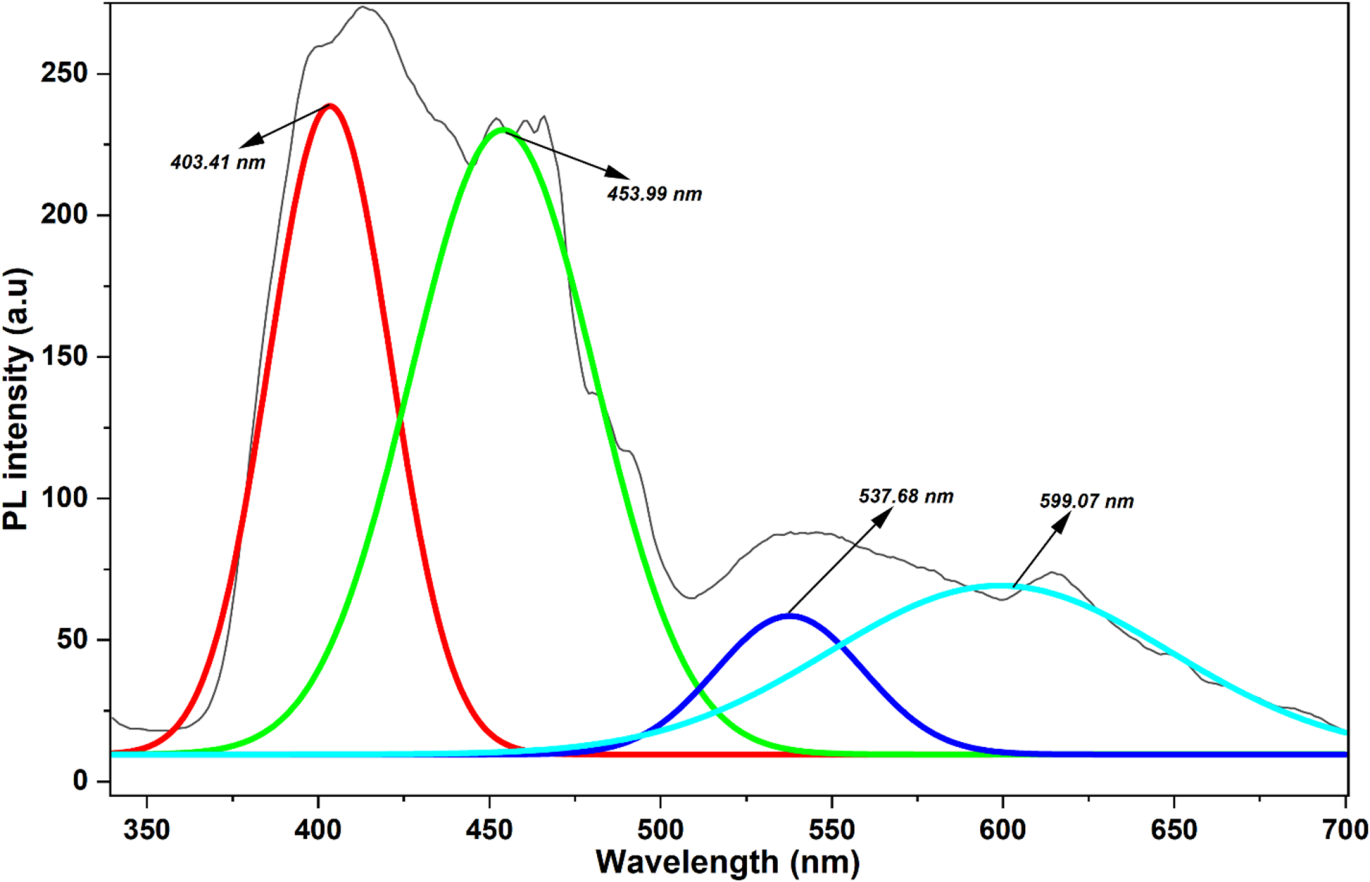
PL spectrum of RFL-ZnO nanoflowers.

PL spectra were collected at ambient conditions using an excitation λ of 325 nm, which corresponds to 3.81 eV. This energy exceeds the ZnO band gap and is sufficient to promote electrons from the valence to the conduction band^48^. As shown in Fig. 6, the spectrum consists of a strong emission in the ultraviolet region and a broader set of bands in the visible region. The deconvoluted UV peak at 403.41 nm (3.07 eV) is allotted to near band edge emission arising from the radiative recombination of free excitons, and reflects the good crystallinity of the RFL-ZnO nanoflowers^49^. The visible emissions at 453.99 nm (2.73 eV), 537.68 nm (2.31 eV), and 599.07 nm (2.07 eV) are commonly associated with intrinsic and surface defects like oxygen vacancies, surface hydroxyl groups, and zinc interstitials^50^. These defect-related states create energy levels inside the band gap, enabling lower energy recombination that yields visible emission, and they also provide trapping sites that facilitate the production of reactive oxygen species during photocatalysis^51^.

### FTIR results

**Fig. 7:**
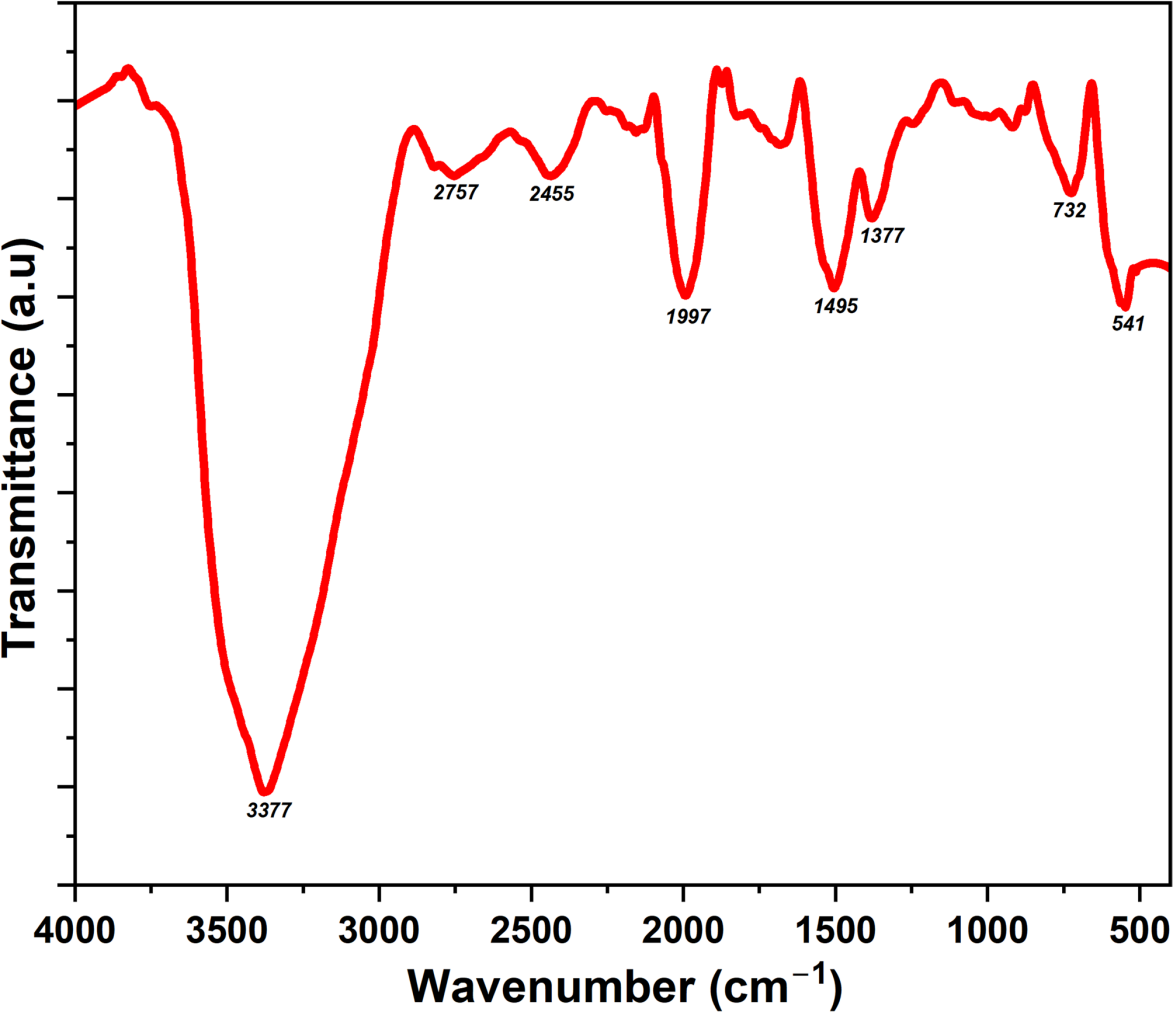
FTIR spectrum of RFL-ZnO nanoflowers.

The FTIR image of the RFL-ZnO nanoflowers in Fig. 7 reveals the functional moieties involved in particle formation and stabilization. A broad high intensity band near 3377 cm^− 1^ is assigned to O-H stretching, arising from hydroxyl groups of phenolic and flavonoid constituents in the RFLE. These hydroxyl functions aid metal precursor reduction and help stabilize ZnO surfaces during green synthesis^52^. The weak 2757 and 2455 cm^− 1^ signals relate to C-H stretching, indicating residual organic moieties from the plant extract^53^. The 1997 cm^− 1^ band belongs to C = O stretching, consistent with carbonyl groups from aldehydes, ketones, or other oxygenated phytochemicals that can act as capping agents^54^. The absorption at 1495 cm^− 1^ reflects C = C stretching of aromatic rings, supporting the role of aromatic species from the extract^55^. The 1377 cm^− 1^ feature corresponds to N = O stretching, suggesting nitrogen-containing groups that may also participate in surface interactions with Zn^2+^ surface sites^56^. The 732 cm^− 1^ signal is due to out-of-plane C-H bending, typical of substituted aromatics or alkenes^57^. Most importantly, the distinct band near 541 cm^− 1^ is a characteristic of Zn-O stretching, confirming the formation of ZnO nanostructures. Thus, the FTIR data show that extract-desrived phytochemicals remain on the ZnO surface as inherent reductants and stabilizers, while the ZnO vibration verifies the presence of ZnO without structural distortion.

### UV-visible monitoring and kinetic study of LVX photodegradation

**Fig. 8:**
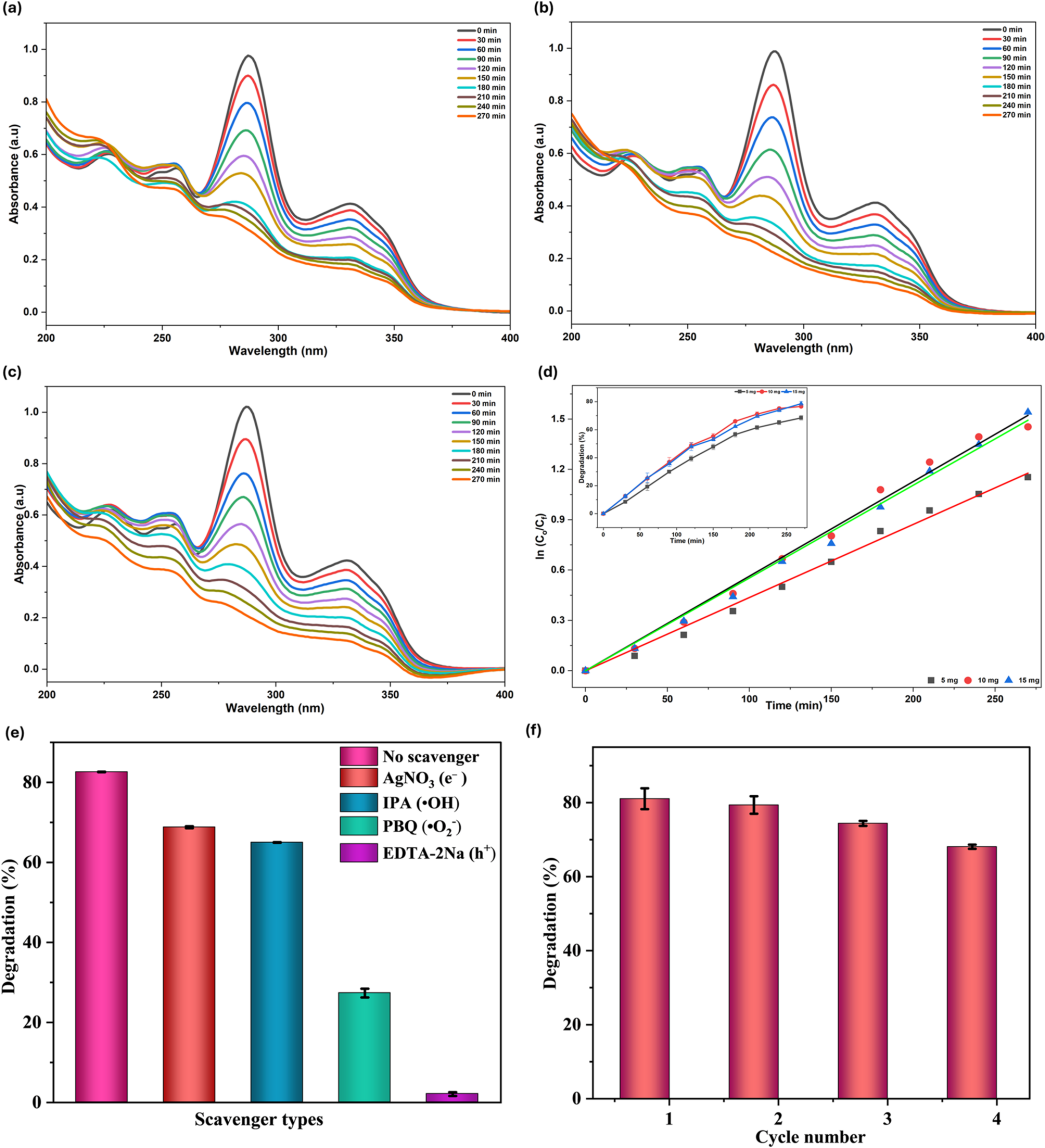
(**a–c**) UV–Vis spectra recorded during LVX degradation over RFL-ZnO nanoflowers at catalyst dosages of 0.05 g/L, 0.10 g/L, and 0.15 g/L, respectively, (**d**) the corresponding pseudo-first-order kinetic plots with inset showing percentage degradation, **(e)** the effect of reactive species scavengers on LVX degradation over RFL-ZnO under natural sunlight, and (**f**) reusability of RFL-ZnO nanoflowers over four successive photocatalytic cycles.

LVX at 30 mg/L was degraded under natural sunlight using RFL-ZnO nanoflowers at 0.05, 0.10, and 0.15 g/L. The process was followed by UV-visible spectra taken at regular intervals, as shown in Fig. 8a–c. LVX shows a clear band at 287 nm from the quinolone chromophore.

When 0.05 g/L of ZnO was used (Fig. 8a), the intensity at 287 nm fell only slowly with irradiation time, consistent with a modest degradation rate and a limited number of active sites and reactive species at this loading. Raising the dosage to 0.10 g/L (Fig. 8b), produced a sharper drop in absorbance, especially early in the run, which signals stronger photocatalytic activity. The most rapid and substantial reduction was obtained at 0.15 g/L (Fig. 8c), consistent with a large number of surface-active sites and more efficient generation of reactive oxygen species that accelerate degradation.

The inset of Fig. 8d shows the percentage removal of LVX versus irradiation time for each catalyst dose. After 270 min of sunlight, the degradation reached 68.4%, 76.6%, and 78.6% at 0.05, 0.10, and 0.15 g/L ZnO, respectively. The steady rise in removal with increasing dose confirms the beneficial effect of catalyst loading on photocatalytic performance^5^. A comparison with previously reported ZnO-based photocatalysts for LVX degradation is provided in Table 1, which further demonstrates that the present RFL-ZnO system exhibits competitive photocatalytic efficiency under sunlight irradiation.

**Table 1:** Comparative performance of reported ZnO-based photocatalysts for LVX degradation.

Sl. no	Catalyst	Photocatalytic condition	Degradation (%)	Refs.
1.	ZnO	Catalyst dose: 20 mg/50 mL, Initial concentration: 10 mg/L, Time: 120 min; UV light	9.4	^63^
2.	ZnO	Catalyst dose: 0.5 g/L, Initial concentration: 10 mg/L, Time: 30 min; visible light	17.8	^64^
3.	ZnO	Catalyst dose: 50 mg/100 mL; Initial concentration: 10 mg/L; Time: 30 min; pH 7; visible light	22.3	^65^
4.	Fe-ZnO	Catalyst dose: 0.5 g/L, Initial concentration: 10 mg/L, Time: 120 min; UVC irradiation	61.25	^66^
5.	ZnO	Catalyst dose: 1.0 g/L; Initial concentration: 5 mg/L; Time: 210 min; Visible light	74.9	^58^
6.	RFL-ZnO	Catalyst dose: 0.15 g/L; Initial concentration: 30 mg/L; Time: 270 min; natural sunlight	78.6	Present study

The kinetic behaviour of the degradation process was analysed using the pseudo-first-order model. The plots between ln(C_0_/C_t_) and time in Fig. 8d are close to linear at all doses, showing that LVX degradation follows pseudo-first-order kinetics. The fitted rate constants were calculated as 0.0044, 0.0055, and 0.0056 min^− 1^ for the dosages of 0.05, 0.10, and 0.15 g/L, respectively, with corresponding R^2^ values exceeding 0.99. These results clearly demonstrate that higher ZnO loadings accelerate the degradation rate by enhancing photon absorption and reactive oxygen species generation^58^.

To identify the main reactive species involved in LVX degradation, radical scavenging experiments were carried out with AgNO_3_, IPA, PBQ, and EDTA-2Na as scavengers for e^–^, •OH, •O_2_^−^, h^+^, respectively (Fig. 8e). In the absence of scavenger, the degradation reached 82.60%. Upon addition of scavengers, the degradation efficiency decreased to 68.79% with AgNO_3_, 64.98% with IPA, 27.34% with PBQ, and only 2.10% with EDTA-2Na. The marked suppression observed in the presence of EDTA-2Na indicates that photogenerated h^+^ played the dominant role in the degradation process. A substantial decrease was also observed with PBQ, confirming the significant contribution of •O_2_^−^ radicals. In contrast, the comparatively smaller reductions caused by AgNO_3_ and IPA suggest that e^–^ and •OH radicals were involved, but to a lesser extent. These results demonstrate that LVX degradation over RFL-ZnO under natural sunlight is governed primarily by h^+^ and •O_2_^−^, while e^–^ and •OH play secondary roles. A related scavenger response has been reported for a recent graphitic carbon nitride/ZnO (g-C3N4/ZnO) photocatalyst, where •O_2_^−^ and h^+^ were likewise identified as the principal reactive species, while e- and •OH exhibited the least contribution to the degradation pathway^59^.

Under sunlight, ZnO absorbs photons with energy at or above its energy band gap, promoting electrons from the valence to the conduction band and generating electron-hole pairs^60^. The holes can directly oxidize LVX and may also react with surface-adsorbed water or hydroxide ions to produce •OH radicals, whereas the conduction band electrons reduce dissolved oxygen to •O_2_⁻ radicals^61^. These reactive species attack the LVX molecules, cleaving the quinolone framework and promoting their conversion into simpler and less harmful products. In addition, residual phytochemicals from the RFLE may contribute to enhanced charge separation by suppressing electron-hole recombination, which further increases the photocatalytic potential of the RFL ZnO nanoflowers under natural sunlight.

### Reusability of RFL-ZnO nanoflowers

The reusability of RFL-ZnO nanoflowers was evaluated over four successive photocatalytic cycles under the optimized conditions, and the corresponding degradation efficiencies are shown in Fig. 8f. The photocatalytic performance decreased gradually from 81.05% in the 1st cycle to 79.36, 74.39, and 68.07% in the 2nd, 3rd, and 4th cycles, respectively. Despite the progressive reduction, the material maintained more than 80% of its initial photocatalytic activity after 4 cycles, implying reasonably good operational stability and reuse potential under natural sunlight. The gradual decrease in performance may be attributed to gradual catalyst deactivation during repeated use, together with agglomeration of the catalyst^62^. Therefore, the results demonstrate that RFL-ZnO nanoflowers possess promising reusability, supporting their potential as a practical green photocatalyst for pharmaceutical wastewater treatment.

## Conclusion

In this work, we synthesized zinc oxide nanoflowers using rubber fig leaf extract and tested them as a photocatalyst to degrade levofloxacin under natural sunlight irradiation. The material shows a distinct flower-like morphology, high phase purity, and surface characteristics that favour light harvesting and charge separation, which together explain its good photocatalytic response. Infrared and X-ray photoelectron spectra reveal contributions from plant-derived species, indicating that phytochemicals take part in nucleation and act as capping reagents that stabilize the nanoparticle surface. Sunlight-driven trials at different catalyst dosages show a clear rise in levofloxacin removal with increasing ZnO loading, with the highest degradation obtained at 0.15 g/L. Kinetic fits follow a pseudo-first-order kinetic model, and the degradation pathway is dominated by reactive oxygen species, mainly hydroxyl and superoxide radicals. Compared with ZnO prepared by conventional chemical routes and with standard treatment options, the rubber fig-mediated nanoflowers provide a cost-effective, low-toxicity, and environmentally benign photocatalyst. Despite these promising outcomes, certain limitations remain. This study is restricted to synthetic wastewater conditions, and further validation in real wastewater matrices is essential. In addition, mineralization efficiency and toxicity of intermediate products were not assessed, and the inclusion of advanced characterization techniques could provide deeper insight into structure-activity relationships. Altogether, the findings support green-synthesized ZnO nanostructures as a realistic option for treating wastewater containing pharmaceutical residues.

## Data Availability

All information necessary to substantiate the findings of this study is fully presented within this manuscript.

## Declarations

### Plant guidelines

All procedures involving plant materials adhere to applicable regulatory and ethical standards.

### Permissions to collect the plant parts/plants

Taxonomic identification and authentication of the plant material were carried out by Dr. Gayathri Pai (Department of Botany, MGM College, Udupi, Karnataka, India). Since the species is common and not protected, collection permits were not necessary, and a herbarium voucher specimen was not prepared.

### Sources of the plant leaves used in this investigation

Information on the plant species and their sources is described in the Methodology part.
